# One Pot Selective Arylation of 2-Bromo-5-Chloro Thiophene; Molecular Structure Investigation via Density Functional Theory (DFT), X-ray Analysis, and Their Biological Activities

**DOI:** 10.3390/ijms17070912

**Published:** 2016-06-28

**Authors:** Nasir Rasool, Aqsa Kanwal, Tehmina Rasheed, Quratulain Ain, Tariq Mahmood, Khurshid Ayub, Muhammad Zubair, Khalid Mohammed Khan, Muhammad Nadeem Arshad, Abdullah M. Asiri, Muhammad Zia-Ul-Haq, Hawa Z. E. Jaafar

**Affiliations:** 1 Department of Chemistry, Government College University Faisalabad, Faisalabad 38000, Pakistan; aqsakanwal.chem@yahoo.com (A.K.); tehmina.rasheed1@gmail.com (T.R.); quratulainayyub@yahoo.com (Q.A.); nr_308@hotmail.com (M.Z.); 2 Department of Chemistry, COMSATS Institute of Information Technology, University Road, Tobe Camp, Abbottabad 22060, Pakistan; mahmood@ciit.net.pk (T.M.); khurshid@ciit.net.pk (K.A.); 3 H.E.J. Research Institute of Chemistry, International Center for Chemical and Biological Sciences, University of Karachi, Karachi 75270, Pakistan; khalid.khan@iccs.edu; 4 Departments of Chemistry, Faculty of Science, King Abdulaziz University, Jeddah 21589, Saudi Arabia; mnachemist@hotmail.com (M.N.A.); aasiri2@kau.edu.sa (A.M.A.); 5 Center of Excellence for Advanced Materials Research (CEAMR), Faculty of Science, King Abdulaziz University, Jeddah 21589, Saudi Arabia; 6 The Patent Office, Karachi 75270, Pakistan; ahirzia@gmail.com; 7 Department of Crop Science, Faculty of Agriculture, 43400 UPM Serdang, Selangor, Malaysia

**Keywords:** 2-bromo-5-chloro thiophenes, Suzuki coupling, density functional theory (DFT), antibacterial, antioxidant

## Abstract

Synthesis of 2,5-bisarylthiophenes was accomplished by sequential Suzuki cross coupling reaction of 2-bromo-5-chloro thiophenes. Density functional theory (DFT) studies were carried out at the B3LYP/6-31G(d, p) level of theory to compare the geometric parameters of 2,5-bisarylthiophenes with those from X-ray diffraction results. The synthesized compounds are screened for *in vitro* bacteria scavenging abilities. At the concentration of 50 and 100 μg/mL, compounds **2b**, **2c**, **2d**, **3c**, and **3f** with IC_50_-values of 51.4, 52.10, 58.0, 56.2, and 56.5 μg/mL respectively, were found most potent against *E. coli.* Among all the synthesized compounds **2a**, **2d**, **3c*,*** and **3e** with the least values of IC_50_ 77, 76.26, 79.13 μg/mL respectively showed significant antioxidant activities. Almost all of the compounds showed good antibacterial activity against *Escherichia coli*, whereas 2-chloro-5-(4-methoxyphenyl) thiophene (**2b**) was found most active among all synthesized compound with an IC_50_ value of 51.4 μg/mL. All of the synthesized compounds were screened for nitric oxide scavenging activity as well. Frontier molecular orbitals (FMOs) and molecular electrostatic potentials of the target compounds were also studied theoretically to account for their relative reactivity

## 1. Introduction

Substituted aromatic compounds are widely synthesized by the well-established family of chemical reactions known as cross-coupling reactions [1,2]. Suzuki cross-coupling reactions generally deliver higher yields under mild reaction conditions, compared to other cross-coupling reactions. Moreover, the boronic acid is commercially available, and the reactions are environmentally friendly [3,4,5,6]. During the last three decades, carbon-carbon coupling for the synthesis of biaryls has replaced classical approaches such as Ullman coupling reactions [7]. Suzuki reaction of tetrabromothiophene is well reported [8,9,10]. The lower cost and easy availability of aryl chlorides made them attractive starting materials in Suzuki–Miyaura reaction with the help of wide varieties of catalytic systems [11,12,13,14].

Thiophene moiety is found to be very potent in various biological activities [15,16,17]. Anti-urease and nitric oxide (NO) scavenging activity of a series of 2-amino-6-arylbenzothiazoles were examined by Gul *et al.* [11]. Various 4-arylthiophene-2-carbaldehydes showed moderate to excellent ability against antibacterial, anti-urease, hemolytic, and antioxidant activities [16]. We became interested in synthesizing unsymmetrical bis-aryl (Ar and Ar′) substituted thiophene by taking the advantage of difference of reactivity of chloro and bromo moiety on thiophene ring. Therefore, in this report, we extend the utilization of aryl chlorides and bromides by reporting the selective Suzuki coupling reactions of 2-bromo-5-chlorothiophene with various electron donating and electron withdrawing aryl boronic acids. These reactions allow efficient synthesis of mono substituted and di-substituted thiophenes by using K_3_PO_4_ as base. However, the compounds **2a**–**c** and **3a**–**c** have already been reported by following different methodologies [18,19,20,21,22], while their biological activities and density functional theory (DFT) studies are being first time reported. After accomplishing the successful synthesis of various mono and di substituted thiopehenes, in continuation of our previous work [23,24], DFT studies were conducted not only to explore the structural properties but also to compare the theoretical structural parameters with those from X-ray diffraction results. Finally, antibacterial and nitric oxide (NO) scavenging activity of the products were investigated.

## 2. Results and Discussion

### 2.1. Preparations

**Scheme 1:**
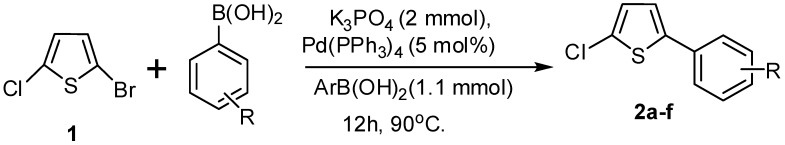
Synthesis of compounds **2a**–**f**. **R**: **2a** = 4-Me, **2b** = 4-MeO, **2c** = 4-Cl, **2d** = 3-Cl,4-F, **2e** = 3,5-dimethyl, and **2f** = 3,4-dichloro.

The 2-aryl-5-chlorothiophenes (**2a**–**f**) were synthesized via Suzuki reaction (Scheme 1) from commercially available (**1**) 2-bromo-5-chloro thiophene (1.0 mmol). The compounds **2a**, **2b** and **2c** have been previously reported but through alternative synthetic strategies [25,26,27,28].

**Figure 1:**
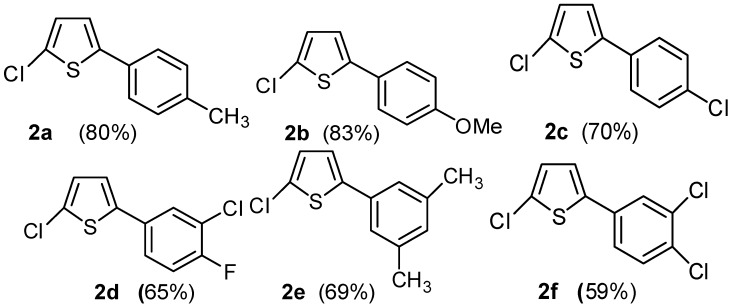
Scope of the compounds **2a**–**f**.

It should be noted that the C–Cl bond strength hampers the reactivity of aryl chlorides, thus, they are reluctant to oxidative addition to Pd(0) [14]. Products **2a**–**f** were prepared following a protocol developed by us [29]. All the products showed moderate to very good yields in the presence of K_3_PO_4_ base (Figure 1). These results suggest that the yield might be sensitive to electron donating and electron withdrawing substituents present on the boronic acid. The base plays a vital role in Suzuki cross-coupling reactions, and it enhances the transmetallation process. Therefore, the choice of the solvent water ratio (3:1.5, 5:1.5 mL) and the quantity of base used are essential to activate boronic acids, and help to obtain good yields.

**Scheme 2:**
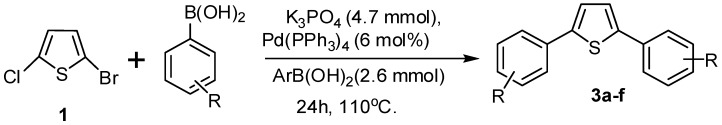
Scope of the compounds of compounds **3a**–**f**. **R**: **3a** = 4-Me, **3b** = 4-MeO, **3c** = 4-Chloro, **3d** = 3-Chloro-4-fluoro, **3e** = 3,5-dimethyl, and **3f** = 3,4-dichloro.

**Figure 2:**
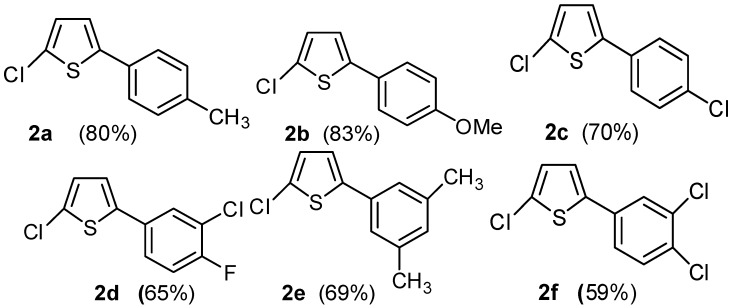
Synthesis of compounds **3a**–**f**.

The biarylthiophenes (**3a**–**f**) having two similar aryl groups were synthesized via Suzuki reaction of **1** (1.0 mmol) with (2.6 equiv.) of several aryl boronic acids (Scheme 2) in moderate to good yields (Figure 2). Reaction conditions such as solvent, temperature, and base played important in controlling the reaction especially for activating chloro functional group to obtain identical di-substituted products. During reaction, temperature was kept at 110 °C. This high temperature plays a significant role for breaking C–Cl bond, and helps in the oxidative addition in Suzuki coupling reactions.

### 2.2. Crystal Structure Determinations

**Figure 3:**
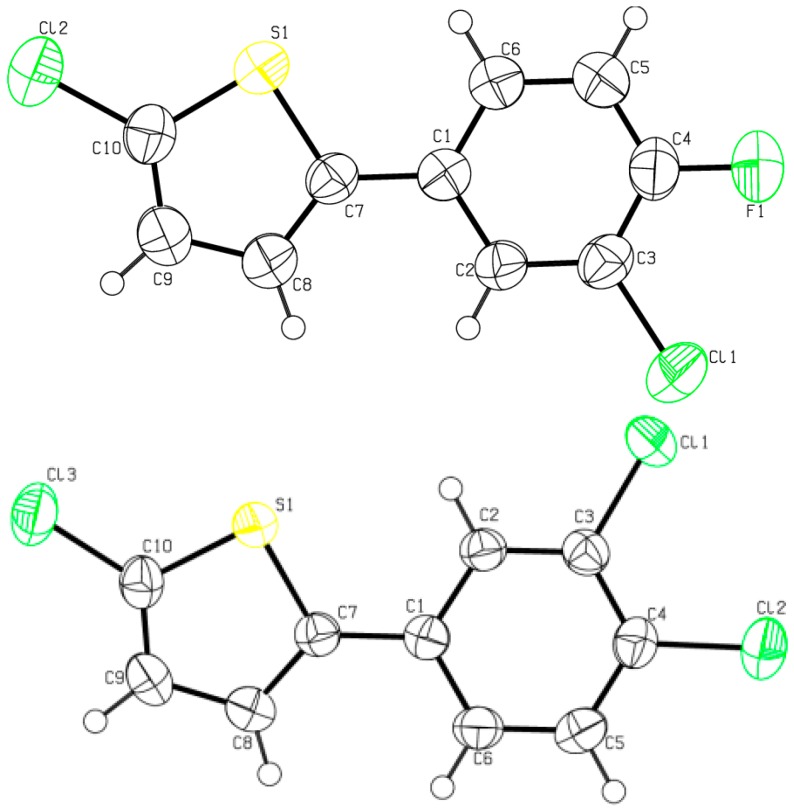
ORTEP plots of **2d** and **2f**.

**Figure 4:**
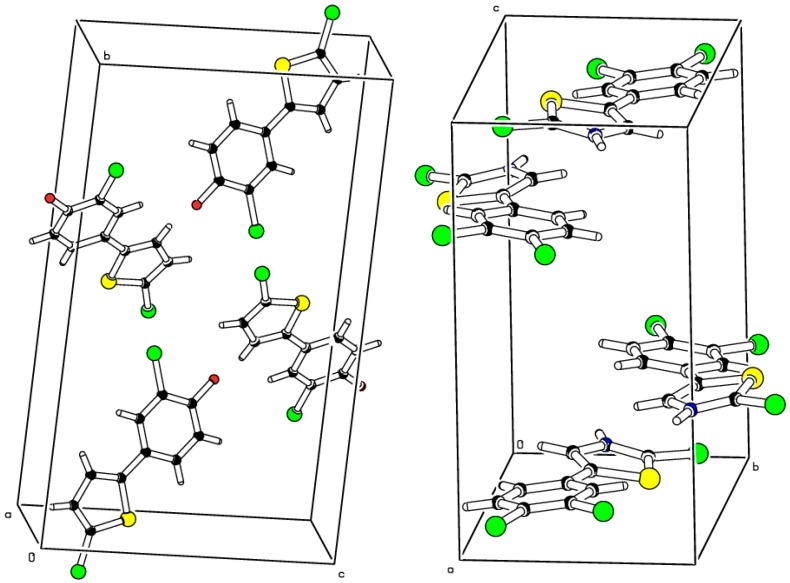
Unit cell diagrams of **2d** and **2f**. (Ball color: green, Cl; red, F; yellow, S; black and blue, C).

Among all synthesized derivatives, suitable crystals were obtained for **2d** and **2f** which were then subjected to X-ray radiation for their structure confirmation and to obtain geometric parameters and spatial interactions. ORTEP plots of both compounds are shown in the Figure 3, and X-ray parameters are being provided in Table 1. The root mean square (RMS) deviation for **2d** is 0.0287 Å, which is indicative of planarity from its fitted atoms with most deviations from Cl2 = −0.0596 (2) Å and C8 = 0.0420 (4) Å. On the other hand, **2f** is not planar with the RMS deviation from the fitted atoms of the molecule at 0.1619 Å, with most deviations from C8 = −0.2746 (2) Å and S1 = 0.2455 (1) Å. The aromatic ring is twisted at a dihedral angle of 11.789° (2) and 2.115° (2) for both, respectively. This also proves the more planarity of **2d**. The C–S–C angles in both molecules are 91.34 and 91.74 degrees, which is in accordance with already reported data [30]. The unit cell diagrams were shown in Figure 4 for compounds I and II, respectively, which does not show any inter- or intra-molecular interactions among the molecules.

### 2.3. Density Functional Theory (DFT) Studies

#### 2.3.1. Molecular Geometries

**Figure 5:**
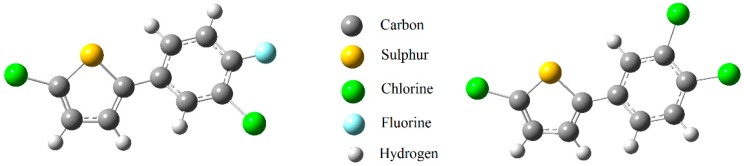
Optimized geometries of **2d** and **2f** at B3LYP/6-31G (d, p) level of DFT.

Molecular geometries obtained through theoretical methods are very useful to explain the three-dimensional structures of compounds, and to compare them with the geometric parameters obtained from X-ray diffraction studies [31]. Among all of the synthesized thiophenes, only **2d** and **2f** gave suitable crystals for X-ray diffraction studies (vide supra). Optimization of all compounds was carried out at B3LYP/6-31G (d, p) level of DFT optimized geometries of **2d** and **2f** are shown in Figure 5, whereas important bonds lengths and bond angles are listed in the Table 2 and Table 3. X-ray geometric parameters of both compounds **2d** and **2f** showed tight correlation with calculated results. The difference in X-ray and calculated bond lengths found in the range 0.003–0.035 Å and 0.002–0.028 Å for **2d** and **2f**, simultaneously. Maximum deviation depicted for C10-S1 in both **2d** and **2f**, which is 0.035 Å and 0.028 Å, respectively (atomic labelling is according to the ORTEP plots shown in Figure 5.

Similarly, the bond angles of both compounds correlated to each other excellently, very minute differences were observed in the range 0.0°–0.9° and 0.1°–1.2° for both compounds **2d** and **2f**, respectively. The maximum difference observed for C5–C4–C3 in **2d**, *i.e.*, 0.9° and for C13-C10-S1 for **2f** (1.2°).

#### 2.3.2. Frontier Molecular Orbital (FMOs) Analysis

FMOs analysis by computational methods is a useful to understand the reactivity and electronic transitions within molecules [32].

**Figure 6:**
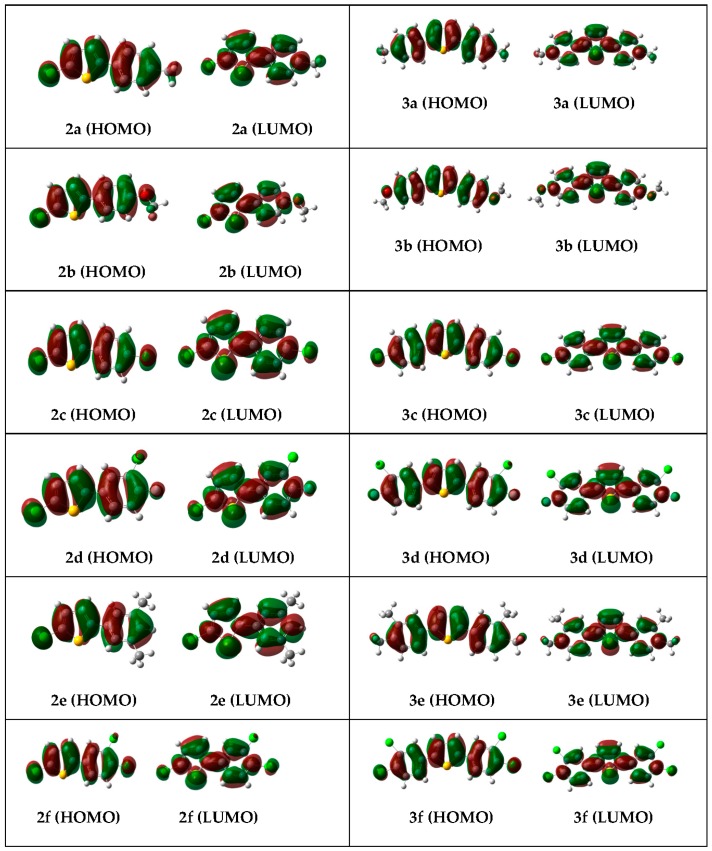
HO/LU orbitals of **2a**–**f** and **3a**–**f**.

Frontier orbitals (HOMO and LUMO), mainly take part in electronic transitions and their energy gap depicts the reactivity [33]. The HOMO-LUMO and electronic properties of compounds (**2a**–**f**) and (**3a**–**f**) were explored at 6-31G (d, p) level of DFT. The distribution patterns of frontier molecular orbitals (HOMOs and LUMOs along with corresponding energies) of all synthesized thiophene derivatives at the ground states have been shown in Figure 6. As reflected from Figure 6, the π cloud in HOMOs and LUMOs of all thiophenes (**2a**–**f**) and (**3a**–**f**) is distributed on the entire skeleton (thiophene and phenyl rings). Introducing the different groups on the benzene ring does not have much effect on the electronic cloud. As reflected form orbital surfaces of compounds **2a**, **2b**, **2c**, **3a**, **3b**, and **3c**, groups attached to the para position of the benzene ring are participating in the π electronic cloud. Whereas the groups attached to the meta position such as in compounds **2d**, **2e**, **3d**, and **3e** are not involved directly in the π electronic cloud.

Detailed HOMO and LUMO energies of all thiophenes along with their gaps are listed in the Table 4. HOMO-LUMO energy difference (E_g_) of mono aryl thiophenes **2a**–**f** is relatively large compare to bis-aryl thiophenes **3a**–**f**. Among all synthesized compounds **3b** and **3f** showed the lowest HOMO-LUMO energy gap *i.e.*, of 3.96 eV and **2e** showed the largest energy gap (4.59 eV).

#### 2.3.3. Molecular Electrostatic Potential (MEP)

Electrostatic potential (ESP) mapping through computer aided methods is very useful parameter to explore the reactivity of organic compounds. Molecular electrostatic potential (MEP) has been applied successfully to understand the enzyme-substrate interactions [34], hydrogen bonding [35], and nuclephilic as well as electrophilic sites in compounds [33].

**Figure 7:**
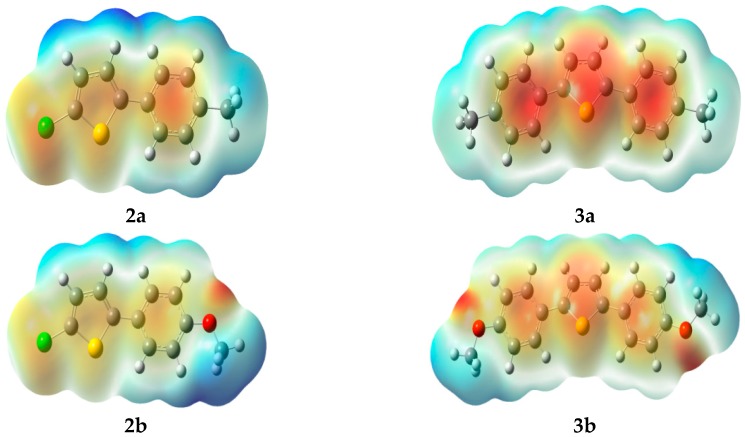

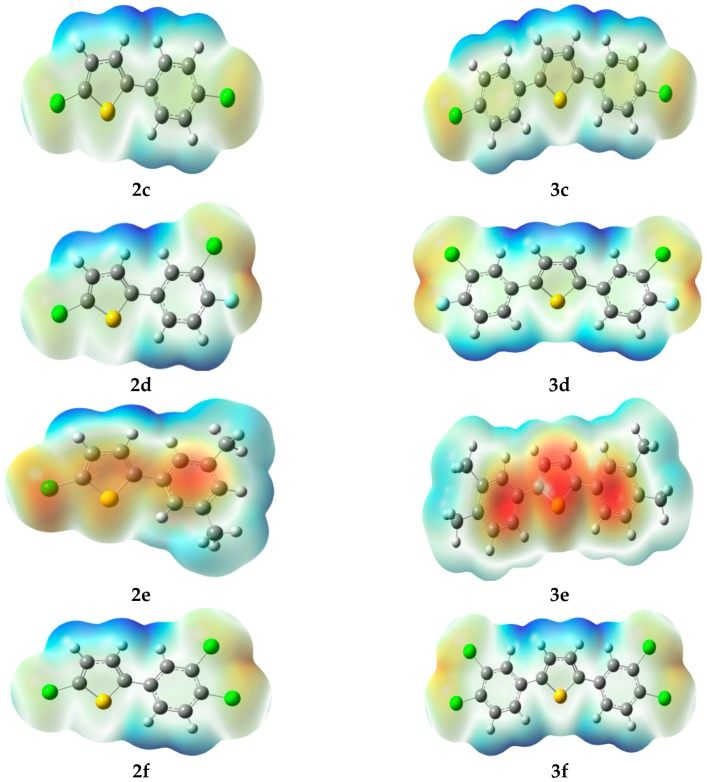
Molecular electrostatic potential (MEP) graphics of mono substituted (**2a**–**f**) and di-substituted (**3a**–**f**) thiophenes (Red = Intense electron rich site, Yellow = Medium electron rich site site, Blue = Electron deficient site, Light green = Almost neutral site, Grey = White = Zero potential).

The nucleophilic, as well as electrophilic, sites in any compound are expressed in term of different color codes, the deep red colour expresses an electron rich site, whereas deep blue expresses an electron-deficient site (Figure 7). From the MEP shown in Figure 7, it is clear that electronic density in **2a** is concentrated on the chloro as well as sulphur atoms of the thiophene ring along with the pi cloud of benzene ring, and protons attached to the thiophene and benzene ring are electron deficient sites. Almost the same trend was observed for **2b** and **3b** but with some extra localization of electronic density on methoxy group oxygen directly attached to benzene. In **2c**, **2d**, **2f**, **3c**, **3d**, and **3f** electronic density was more dispersed and concentrated on chloro and floro groups, due to their electron withdrawing nature and positive potential is concentrated on the protons attached to the thiophene and benzene rings. Compounds **2e** and **3e** are bearing the electron-donating methyl groups attached to the rings; therefore, the electronic density is localized on the pi cloud of both the thiophene and benzene rings.

## 3. Biological Studies

### 3.1. Antibacterial Activity

**Figure 8:**
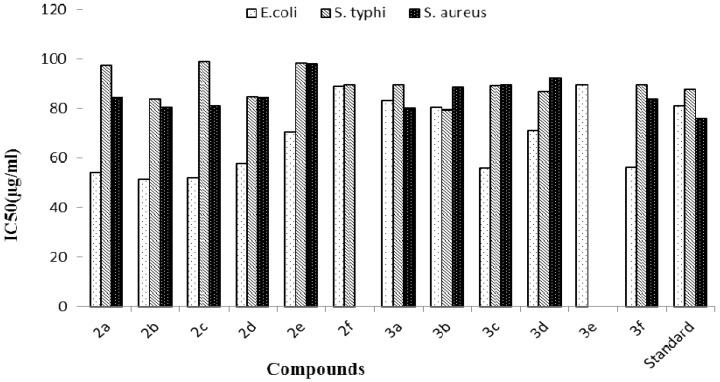
IC_50_ values of antibacterial activity.

Antibacterial activity is related to the existence of some elements in a compound, such as sulfur [8,36,37,38]. Recently, benzothiophene derivatives have been used in many therapies [39]. The newly-synthesized thiophene molecules **2a** to **3f** were tested against several strains of Gram-negative bacteria (*Eschericha coli*, *Shigella dysenteriae*, *Pseudomonas aeruginosa* and *Salmonella typhi*) and Gram-positive bacteria (*Staphylococcus aureus* and *Bacillus subtilis*). Ampicillin was used as a standard drug, and all data are shown in Figure 8. Electron-withdrawing and electron-donating substituents have great effect on antibacterial activity of synthesized compounds [38]. At the concentration of 50 and 100 μg/mL, compounds **2c**, **2d**, **3c**, and **3f** (containing electron-withdrawing groups) with an IC_50_ value of 52.10, 58.0, 56.2, and 56.5 μg/mL, respectively, were found most potent against *E. coli*. Surprisingly, it has been observed that **2b** exhibited highest bacterial inhibition activity with an IC_50_ value of 51.4 μg/mL and **2a** also showed unexpected IC_50_ value of 54.17 μg/mL, and both of these compounds contain electron-donating groups. Compounds **2e** and **3d** exhibited significant activity with an IC_50_ value of 70.5 and 71.2 μg/mL. However **3b** showed IC_50_ value almost equal to standard Ampicillin while the remaining compounds exhibited IC_50_ values more than the standard against *E. coli* and were found less active.

Compounds **2b**, **2d**, and **3b** with an IC_50_ value of 80.0, 80.9, and 79.52 μg/mL, respectively, showed moderate activity against *S. typhi*. However, **3d** exhibited IC_50_ value nearly equal to the standard while all other compounds were found less active than the standard having a high value of IC_50_ as compared to the standard.

### 3.2. Antioxidant Activity

Inflammatory disorders in the human body are associated with nitric oxide (NO). From the reported data, it is observed that various thiophene derivatives exhibit antioxidant activity [39,40,41,42] and can also be used as antitumor agent [43].

**Figure 9:**
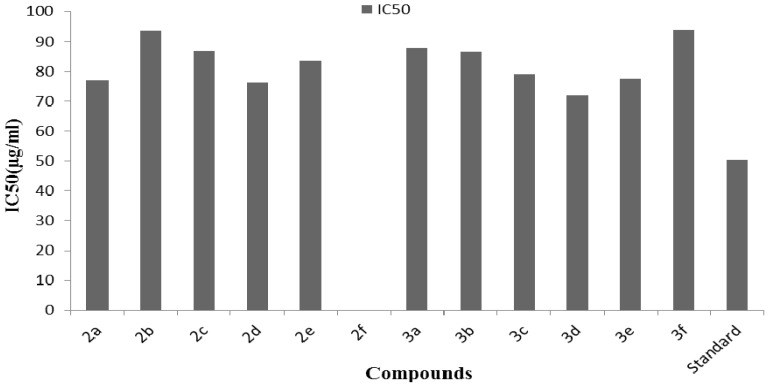
IC_50_ values of nitric oxide scavenging activity.

The antioxidant activity of compounds **2a**–**3f** was tested by nitric oxide scavenging activity method and the results were compared with that of standard natural antioxidant ascorbic acid. As shown in Figure 9, almost all the synthesized compounds showed radical scavenging activity, but the highest scavenger activity was observed in the compound **3d** whose IC_50_ value was 72. Among all the synthesized compounds **2a**, **2d**, **3c**, and **3e** with the least values of IC_50_ 77, 76.26, 79.13 and 77.4 μg/mL, respectively, showed significant antioxidant activities. Moderate nitric oxide scavenging activity was observed in all the remaining compounds, except **2f**, which is found inactive against this activity.

## 4. Materials and Methods

A Bruker ARX 600 MHz FT-NMR spectrometer (Billerica, MA, USA) was used to study. NMR spectra were taken on a Bruker ARX 600 MHz FT-NMR spectrometer while relishing deuterated CDCl_3_ as internal reference.

### 4.1. Synthesis of 2-Aryl-5-chloro thiophenes (**2a**–**f**)

To a stirred solution of 3 mL dioxane of 2-bromo-5-chlorothiophene (1.0 mmol) and tetrakis(triphenylphosphine)palladium(0) (5.0 mol %) were added and stirred for a period of 30 min. To this mixture was added Ar-B(OH)_2_ (1.1 mmol),water (1.5 mL) and 2-mmol of K_3_PO_4_. The mixture was stirred at 90 °C for a period of 12 h. With the help of column chromatography the resultant product was purified.

#### 4.1.1. 2-Chloro-5-(4-methylphenyl) thiophene (**2a**)

Pale yellow solid, mp. 168 °C, ^1^H-NMR: δ = 7.38 (d, *J* = 7.9, 2H-Ar), 7.16 (d, *J* = 8, 2H-Ar), 7.00 (d, *J* = 4, 1H-Thiophene), 6.85 (d, *J* = 3.6, 1H Thiophene), 2.36 (s, 3H-CH_3_). ^13^C-NMR: δ = 21.2 (CH_3_ of aryl), 125.7, 127.1, 127.4, 129.8, 130.2, 132.0, 138.9. EIMS *m*/*z*: 208.71; [M + H^+^]:[M − Cl]^+^ = 172.26; [M − CH_3_ and benzene]^+^ = 91.14. Anal.(%) calcd for C_11_H_9_ClS, C 63.60, H 4.35; found C 63.66, H 4.31.

#### 4.1.2. 2-Chloro-5-(4-methoxyphenyl) thiophene (**2b**)

Light green solid, mp. 172 °C; ^1^H-NMR: δ = 7.40 (d, *J* = 8.3, 2H-Ar), 7.01 (d, *J* = 3.6, 1H-Thiophene), 6.90 (d, *J* = 8.8, 2 H-Ar), 6.85 (d, *J* = 3.8, 1H Thiophene) 3.81 (s, 3 OCH_3_). ^13^C-NMR: δ = 56.1(OCH_3_ of aryl), 115.0, 125.8, 126.3, 127.3, 127.9, 139.5, 160.9. EIMS *m*/*z*: 224.71; [M + H]^+^:[M − OMe]^+^ = 194.68; [M − Cl]^+^ = 190.26. Anal.(%) calcd for C_11_H_9_ClOS, C 58.80; H 4.04; found C 58.20, H 4.10.

#### 4.1.3. 2-Chloro-5-(4-chlorophenyl) thiophene (**2c**)

Yellowish green solid, mp. 180 °C; ^1^H-NMR: δ = 7.42 (d, *J* = 7.2, 2H-Ar), 7.35 (d, *J* = 8, 2H-Ar), 7.10 (d, *J* = 3.5, 1H Thiophene), 6.89 (d, *J* = 3.8, 1H Thiophene). ^13^C-NMR: δ = 125.9, 127.1, 128.4, 129.9, 132.0, 134.6, 139.1. EIMS *m*/*z*: 229.13; [M + H]^+^:[M − Cl]^+^ = 194.68; [M − 2Cl]^+^ = 160.24. Anal.(%) calcd for C_10_H_6_Cl_2_S, C 52.46, H 2.69; found C 52.42 ; H 2.64. EIMS (*m*/*z*, +ion mode).

#### 4.1.4. 2-Chloro-5-(3-chloro-4-fluorophenyl) thiophene (**2d**)

Yellow solid, mp. 185 °C; ^1^H-NMR: δ = 7.32(m, 3H-Ar), 6.98 (d, *J* = 4, 1H-Thiophene), 6.87 (d, *J* = 3.8, 1H-Thiophene). ^13^C-NMR: δ = 117.8, 121.3, 126.0, 126.8, 127.6, 128.8, 130.1, 138.9, 158.2. EIMS *m*/*z*: 247.12; [M + H^+^]:[M − F and Cl]^+^ = 192.68. Anal.(%) calcd for C_10_H_5_Cl_2_FS, C 48.58; H 2.08, found C 48.60; H 2.04.

#### 4.1.5. 2-Chloro-5-(3,5-dimethylphenyl) thiophene (**2e**)

Greenish yellow solid, mp. 166 °C; ^1^H-NMR: δ = 7.30–7.19 (m, 3H-Ar), 7.01 (d, *J* = 3.5, 1H-Thiophene), 6.99 (d, *J* = 3.7, 1H, Thiophene), 3.27 (s, 6H-CH_3_). ^13^C-NMR: δ = 21.8 (CH_3_ of aryl), 125.1, 126.1, 126.6, 127.5, 128.1, 128.9, 131.3, 138.8. EIMS *m*/*z*: 222.73; [M + H^+^]:[M − Br and 2CH_3_]^+^ = 157.24. Anal.(%) calcd for C_12_H_11_ClS, C 64.71, H 4.98, found C 64.77; H 4.93.

#### 4.1.6. 2-Chloro-5-(3,4-dichlorophenyl) thiophene (**2f**)

Brownish yellow solid, mp. 186 °C; ^1^H-NMR: δ = 7.47–7.30 (m, 3H-aryl) 7.20 (d, *J* = 3.8, 1H Thiophene), 7.01 (d, *J* = 3.3, 1H Thiophene). ^13^C-NMR: δ = 126.2, 127.2, 127.7, 128.4, 130.3, 132.4, 133.6, 133.9, 139.1. EIMS *m*/*z*: 263.57; [M + H^+^]:[M − 3Cl]^+^ = 157.24; [M − 2Cl and benzene]^+^ = 117.57. Anal.(%) calcd for C_10_H_5_Cl_3_S, C 45.52, H 1.89, found C 45.57; H 1.91.

### 4.2. Synthesis of Biarylthiophenes (**3a**–**f**)

Stirred solution of **1** (1.0 mmol) and tetrakis(triphenylphosphine)palladium(0) (6.0 mol %) were added and stirred for a period of 30 min. To this mixture was added Ar-B(OH)_2_ (2.6 mmol),water (1.5 mL) and 4.7-mmol of K_3_PO_4_. The mixture was stirred at 90 °C for a period of 12 h. The mixture was stirred at 110 °C for a period of 24 h. With the help of column chromatography the resultant product was purified.

#### 4.2.1. 2,5-Bis(4-methylphenyl) thiophene (**3a**)

Pale yellow solid, mp. 191 °C; ^1^H-NMR: δ = 7.05 (s, 2H-thiophene), 7.39–7.20 (m, 8H-Ar), 3.02 (s, 6H-CH_3_). ^13^C-NMR: δ = 21.1 (CH_3_ of aryl), 125.7, 128.6, 129.8, 130.9, 131.2, 137.4, 138.1. EIMS *m*/*z*: 264.38 [M + H^+^]:[M − 2CH_3_]^+^ = 234.33. Anal.(%) calcd for C_18_H_16_S, C 81.77, H 6.10, found C 81.71; H 6.17.

#### 4.2.2. 2,5-Bis(4-methoxyphenyl) thiophene (**3b**)

Greenish white solid, mp. 177 °C; ^1^H-NMR: δ = 7.07 (s, 2H-Thiophene), 7.33–7.30 (m, 8H-Ar), 3.79 (s, 6H-OCH_3_). ^13^C-NMR: δ = 55.4 (OCH_3_ of aryl), 115.2, 125.9, 128.6, 137.4, 160.8. EIMS *m*/*z*: 296.28 [M + H^+^]:[M − OCH_3_]^+^ = 265.36; [M − OCH_3_ and benzene]^+^ = 189.26. Anal.(%) calcd for C_18_H_16_O_2_S, C 79.91, H 5.40, found C 79.94; H 5.44.

#### 4.2.3. 2,5-Bis(4-chlorophenyl) thiophene (**3c**)

Yellow solid, mp. 196 °C; ^1^H-NMR: δ = 7.05 (s, 2H-Thiophene), 7.41–7.30 (m, 8H-Ar). ^13^C-NMR: δ = 128.2, 129.5, 130.9, 134.6, 137.3. EIMS *m*/*z*: 305.21 [M + H]^+^:[M − 2Cl]^+^ = 234.31:[M − Cl and benzene]^+^ = 193.76. Anal.(%) calcd for C_18_H_16_O_2_S, C 62.90, H 3.38, found C 62.96; H 3.30.

#### 4.2.4. 2,5-Bis(3-chloro-4-fluorophenyl) thiophene (**3d**)

Yellow crystals, mp. 183 °C; ^1^H-NMR: δ = 7.20 (s, 2H-Thiophene), 7.69–7.58 (m, 6H-Ar). ^13^C-NMR: δ = 117.6, 121.0, 124.8, 126.9, 128.8, 129.2, 130.4, 131.3, 133.8, 137.6, 159.1, 163.4. EIMS *m*/*z*: 341.20 [M + H^+^]:[M − 2Cl]^+^ = 270.31; [M − 2F]^+^ = 303.32. Anal.(%) calcd for C_16_H_8_Cl_2_F_2_S, C 56.32; H 2.36, found C 56.38; H 2.38.

#### 4.2.5. 2,5-Bis(3,5-dimethylphenyl) thiophene (**3e**)

Greenish yellow solid, mp. 185 °C; ^1^H-NMR: δ = 7.02 (s, 2H-Thiophene), 7.54–7.47 (m, 6H-Ar), 3.29 (s, 12H-CH_3_). ^13^C-NMR: δ = 21.7 (CH_3_of aryl), 127.8, 128.8, 131.1, 133.8, 138.2, 138.6, 139.1. EIMS *m*/*z*: 292.44 [M + H^+^]; [M − 4Me]^+^ = 232.34**.** Anal.(%) calcd for C_20_H_20_S, C 82.14, H 6.89, found C 82.18; H 6.80.

#### 4.2.6. 2,5-Bis(3,4-dichlorophenyl) thiophene (**3f**)

Golden yellow solid, mp. 170 °C; ^1^H-NMR: δ = 7.15 (s, 2H-Thiophene), 7.58–7.53 (m, 6H-Ar). ^13^C-NMR: δ = 126.9, 128.3, 129.1, 130.4, 132.9, 133.6, 138.2. EIMS *m*/*z*: 373.22 [M + H^+^]:[M − 4Cl]^+^ = 232.33; [M − 2Cl]^+^ = 303.22. Anal.(%) calcd forC_16_H_8_Cl_4_S, C 51.30, H 2.18, found C 51.37; H 2.16.

### 4.3. X-ray Diffraction Analysis

Single crystals of both thiophenes **2d** and **2f** with appropriate sizes were chosen from available sample under microscope. Which were fixed on glass tip using glue, purchased from local market. The glass needle was supported by copper pin and magnetic base. This whole assembly was mounted on Agilent SuperNova (dual source) Agilent Technologies Diffractometer, equipped with graphite-monochromatic Cu/Mo Kα radiation for data collection. The data collection was accomplished using CrysAlisPro software [44], at 296 K under Cu Kα radiation. The structures were solved using SHELXS-97 [45], and refined by full-matrix least-squares methods on F^2^ using SHELXL-97, in-built with X-Seed [46]. All non–hydrogen atoms were refined anisotropically by full–matrix least squares methods [45]. The figures were drawn using PLATON in-built with wingx.

There are only aromatic (C–H) hydrogen atoms, which were positioned geometrically and treated as riding atoms with C–H = 0.93 Å and Uiso(H) = 1.2 Ueq(C) carbon atoms.

The CIFs for both molecules have been submitted to (The Cambridge Crystallographic Data Centre) CCDC and got CCDC numbers 1469610 and 1469611 for molecule **2d** and **2f** respectively. These CIFs can be ordered free of cost from CCDC 12 Union Road, Cambridge CB21 EZ, UK.

### 4.4. Computational Methods

Theoretical investigations were performed by using Gaussian 09 software [47]. Visualizations of graphics/geometries was achieved by using Gauss view 05 program [48]. Geometries of (**2a**–**f**) and (**3a**–**f**) were optimized by adopting hybrid B3LYP method without any symmetry constraints along with 6-31G (d, p) basis set at DFT level of theory [49,50]. Frontier molecular orbital analysis and molecular electrostatic potential mapping of both series (**2a**–**f**) and (**3a**–**f**) were simulated at same level of DFT as used for energy minima optimization.

### 4.5. Antibacterial Assay

The antibacterial assay of compounds **2a**–**3f** was accomplished by method reported by of Nasrullah and co-workers [12]. *Bacillus subtilis*, *Staphylococcus aureus* were used as Gram-positive bacteria and *Escherichia coli*, *Pseudomonas aeruginosa*, *Salmonella typhi*, *Shigelladysenteriae* used as Gram-negative bacteria.

Solutions of the compounds were made by dissolving in a solvent of known concentration (5 and 10 μg/mL). Samples of different concentrations were prepared by already known volumes of compounds. Ampicillin (positive control) was prepared by using the same methodology. By using only solvent negative control was prepared. At 137 °C for 30 min glass apparatus was sterilized. In sterile glass Petri plates nutrient agar was added. In test tubes having nutrient broth, sub-cultures were injected and left at 37 °C for 16 h on rotary shaker. On inoculated nutrient agar medium positive and negative controls, all discs and test samples were solidified at 37 ± 2 °C for 24 h. With the help of an ordinary ruler, microbial growth was measured.

### 4.6. Nitric Oxide Scavenging Activity

By following procedure reported by Garrat and co-workers [51] nitric oxide scavenging activity of all compounds was carried out.

## 5. Conclusions

In summary, we report the synthesis of various 2-aryl-5-chlorothiophenes and 2,5-biarylthiophenes, starting from 2-bromo-5-chloro thiophenes. In Suzuki coupling reactions, different boronic acids/esters react with 2-bromo-5-chloro thiophenes in the presence of a palladium catalyst. X-ray and calculated geometric parameters of **2d** and **2f**, corroborate very nicely to each other. Reactive sites and electronic effect of group attached to benzene ring was investigated by ESP analysis. By noting the results of this study it is revealed that some of the synthesized compounds of 2-bromo-5-chloro thiophenes can be used as antibacterial agents.

## Figures and Tables

**Table 1:** Crystal data and structure refinement parameters for compounds **2d** and **2f**.

Compound	2d	2f
Empirical formula	C_10_H_5_Cl_2_SF	C_10_H_5_SCl_3_
Formula weight	247.10	263.55
Temperature/K	293(2)	293(2)
Crystal system	monoclinic	monoclinic
Space group	P2_1_/c	P2_1_/n
a/Å	3.8805(3)	11.3539(3)
b/Å	21.8785(12)	6.9384(2)
c/Å	11.9541(8)	13.1916(4)
α/°	90	90
β/°	98.688(8)	90.807(3)
γ/°	90	90
Volume/Å^3^	1003.25(12)	1039.10(5)
Z	4	4
ρ_calc_g/cm^3^	1.636	1.685
μ/mm^−1^	7.516	9.467
F(000)	496.0	528.0
Crystal size/mm^3^	0.26 × 0.06 × 0.06	0.33 × 0.18 × 0.15
Radiation	CuKα (λ = 1.54184)	CuKα (λ = 1.54184)
2θ range for data collection/°	8.082 to 162.368	10.208 to 152.446
Index ranges	−4 ≤ h ≤ 4, −27 ≤ k ≤ 25, −12 ≤ l ≤ 14	−11 ≤ h ≤ 14, −8 ≤ k ≤ 8, −16 ≤ l ≤ 16
Reflections collected	6585	10801
Independent reflections	2064 (R_int_ = 0.0292, R_sigma_ = 0.0228)	2170 (R_int_ = 0.0324, R_sigma_ = 0.0183)
Data/restraints/parameters	2064/0/127	2170/0/127
Goodness-of-fit on F^2^	0.981	1.015
Final R indexes (I ≥ 2σ (I))	R_1_ = 0.0463, wR_2_ = 0.1180	R_1_ = 0.0303, wR_2_ = 0.0795
Final R indexes (all data)	R_1_ = 0.0643, wR_2_ = 0.1466	R_1_ = 0.0340, wR_2_ = 0.0830
Largest diff. peak/hole/e Å^−3^	0.37/−0.41	0.27/−0.30

**Table 2:** Some selected X-ray and simulated bond lengths (Å) of **2d** and **2f** (atomic labels are with reference to *ORTEP* plot Figure 3).

(2d)	X-ray	Calc. (B3LYP)	(2f)	X-ray	Calc. (B3LYP)
C1–C2	1.400 (4)	1.406	C1–C2	1.399 (2)	1.405
C1–C6	1.391 (5)	1.406	C1–C6	1.401 (2)	1.404
C1–C7	1.474 (4)	1.467	C1–C7	1.464 (2)	1.466
C2–C3	1.375 (5)	1.39	C2–C3	1.374 (2)	1.389
C3–C4	1.373 (5)	1.396	C3–C4	1.387 (3)	1.396
C3–Cl1	1.727 (4)	1.746	C3–Cl1	1.728 (18)	1.747
C4–C5	1.363 (6)	1.389	C4–C5	1.386 (3)	1.401
C5–C6	1.371 (5)	1.391	C4–Cl2	1.726 (18)	1.745
C7–C8	1.355 (5)	1.374	C5–C6	1.374 (3)	1.392
C7–S1	1.725 (3)	1.757	C7–C8	1.369 (2)	1.375
C8–C9	1.421 (5)	1.424	C7–S1	1.734 (18)	1.757
C9–C10	1.342 (6)	1.366	C8–C9	1.415 (3)	1.423
C10–S1	1.707 (4)	1.742	C9–C10	1.346 (3)	1.366
C10–Cl2	1.715 (4)	1.73	C10–S1	1.714 (18)	1.742
C4–F1	1.357 (4)	1.34	C10–Cl3	1.714 (2)	1.73

**Table 3:** Some selected X-ray and simulated bond angles (°) of **2d** and **2f** (atomic labels are with Reference to Figure 3).

Bond (2d)	X-ray	Calc. (B3LYP)	Bond (2f)	X-ray	Calc. (B3LYP)
C2–C1–C6	118.0 (3)	118.4	C2–C1–C6	117.7 (16)	118.1
C2–C1–C7	120.3 (3)	119.7	C2–C1–C7	121.1 (16)	121.4
C6–C1–C7	121.7 (3)	121.8	C6–C1–C7	121.0 (16)	120.4
C3–C2–C1	120.3 (3)	120.7	C3–C2–C1	121.1 (17)	121.1
C2–C3–C4	119.7 (3)	119.7	C2–C3–C4	120.1 (17)	120
C2–C3–Cl1	120.9 (3)	120.5	C2–C3–Cl1	118.8 (15)	118.5
C4–C3–Cl1	119.5 (3)	119.6	C4–C3–Cl1	120.9 (14)	121.4
C5–C4–C3	121.3 (3)	120.4	C3–C4–C12	121.1 (15)	121.7
C6–C5–C4	119.4 (4)	119.6	C5–C4–C3	119.5 (17)	119.1
F1–C4–C3	119.1 (4)	119.9	C5–C4–C12	119.2 (15)	119
C5–C4–F1	119.6 (4)	119.5	C6–C5–C4	120.3 (18)	120.6
C5–C6–C1	121.2 (3)	120.9	C5–C6–C1	120.9 (17)	120.8
C1–C7–S1	120.6 (2)	121.1	C1–C7–S1	120.4 (13)	121.3
C8–C7–C1	129.2 (3)	128.5	C8–C7–C1	129.4 (17)	128.3
C8–C7–S1	110.3 (3)	110.3	C8–C7–S1	110.0 (14)	110.3
C7–C8–C9	113.7 (4)	114.1	C7–C8–C9	113.9 (17)	114.1
C10–C9–C8	111.8 (4)	111.9	C10–C9–C8	111.6 (17)	111.9
C9–C10–S1	112.5 (3)	112.4	C9–C10–S1	112.9 (15)	112.4
C10–S1–C7	91.74 (17)	91.1	C9–C10–Cl3	127.7 (15)	127
S1–C10–C12	120.4 (2)	120.5	Cl3–C10–S1	119.2 (12)	120.4
C9–C10–C12	127.1 (3)	127	C10–S1–C7	91.34 (9)	91

**Table 4:** HOMO and LUMO energies along with energy gaps.

Entry No.	HOMO (eV)	LUMO (eV)	HOMO-LUMO (ΔE) eV
**2a**	−5.71	−1.14	4.57
**2b**	−5.49	−1.02	4.47
**2c**	−5.96	−1.46	4.50
**2d**	−6.07	−1.48	4.59
**2e**	−5.72	−1.13	4.59
**2f**	−6.13	−1.66	4.47
**3a**	−5.24	−1.21	4.03
**3b**	−4.97	−1.01	3.96
**3c**	−5.68	−1.70	3.98
**3d**	−5.84	−1.76	4.08
**3e**	−5.16	−1.14	4.02
**3f**	−5.96	−1.99	3.97
